# Search and Graph Database Technologies for Biomedical Semantic Indexing: Experimental Analysis

**DOI:** 10.2196/medinform.7059

**Published:** 2017-12-01

**Authors:** Isabel Segura Bedmar, Paloma Martínez, Adrián Carruana Martín

**Affiliations:** ^1^ LaBDA Group Department of Computer Science Universidad Carlos III de Madrid Leganés Spain

**Keywords:** information storage and retrieval, semantic indexing, Medical Subject Headings

## Abstract

**Background:**

Biomedical semantic indexing is a very useful support tool for human curators in their efforts for indexing and cataloging the biomedical literature.

**Objective:**

The aim of this study was to describe a system to automatically assign Medical Subject Headings (MeSH) to biomedical articles from MEDLINE.

**Methods:**

Our approach relies on the assumption that similar documents should be classified by similar MeSH terms. Although previous work has already exploited the document similarity by using a k-nearest neighbors algorithm, we represent documents as document vectors by search engine indexing and then compute the similarity between documents using cosine similarity. Once the most similar documents for a given input document are retrieved, we rank their MeSH terms to choose the most suitable set for the input document. To do this, we define a scoring function that takes into account the frequency of the term into the set of retrieved documents and the similarity between the input document and each retrieved document. In addition, we implement guidelines proposed by human curators to annotate MEDLINE articles; in particular, the heuristic that says if 3 MeSH terms are proposed to classify an article and they share the same ancestor, they should be replaced by this ancestor. The representation of the MeSH thesaurus as a graph database allows us to employ graph search algorithms to quickly and easily capture hierarchical relationships such as the lowest common ancestor between terms.

**Results:**

Our experiments show promising results with an F1 of 69% on the test dataset.

**Conclusions:**

To the best of our knowledge, this is the first work that combines search and graph database technologies for the task of biomedical semantic indexing. Due to its horizontal scalability, ElasticSearch becomes a real solution to index large collections of documents (such as the bibliographic database MEDLINE). Moreover, the use of graph search algorithms for accessing MeSH information could provide a support tool for cataloging MEDLINE abstracts in real time.

## Introduction

### Biomedical Semantic Indexing

The last two decades have witnessed tremendous advances in our knowledge of life sciences and medicine, leading to an exponential growth of the biomedical literature. There are several biomedical bibliographic databases such as EMBASE, OVID, Ebsco Host Research databases, Scielo, Cochrane, and the largest one, with 5600 journals and over 26 million articles, MEDLINE. In 2015, more than 806,000 citations were added to MEDLINE with a load of 2000 to 4000 documents per day. This quickly growing volume of articles is an overwhelming challenge that requires a very specialized knowledge for organizing this bibliographic database.

To support the classification and indexing of the content of the MEDLINE database, the US National Library of Medicine (NLM) produces and maintains a thesaurus of medical concepts, MeSH (Medical Subject Headings), which is reviewed and updated continually (eg, 310 new headings were added to MeSH in 2015). Each document in MEDLINE is represented with a set of MeSH terms that describe its subject topic. This task, which is generally known as biomedical semantic indexing, is a crucial task to facilitate literature search because MeSH terms can be used in search queries to retrieve references that were annotated with these terms or with their hierarchically related terms in MeSH (ie, their synonyms, hypernyms, or hyponyms). The task of identifying the MeSH terms that best represent a MEDLINE article is manually performed by human experts (so-called curators). NLM also provides some basic principles [1] to assign MeSH terms that curators should follow when they catalog articles.

Biomedical semantic indexing is usually a costly, time-consuming, and laborious task [2]. Therefore, there is an urgent need to explore semiautomatic methods to support semantic indexing.

**Figure 1:**
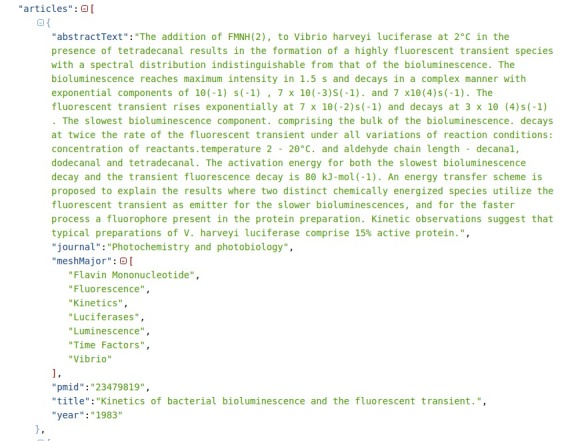
JSON-based format for the training data in the biomedical semantic indexing and question answering challenge BioASQ task 4a.

Several challenges such as Critical Assessment of Information Extraction in Biology (BioCreative) [3], Workshop on Biomedical Natural Language Processing (BioNLP) shared tasks [4,5], Informatics for Integrating Biology & the Bedside (i2b2) [6], and DDIExtraction [7,8] have significantly contributed to improve and advance the state of the art in Natural Language Processing for biomedicine, especially in the information extraction task. Similarly, the biomedical semantic indexing and question answering challenge (BioASQ) is being organized since 2013 to encourage and promote research in these fields and provide a common framework for assessment. The objective of the task is to tag an article with a set of terms (also known as headings or descriptors) from the MeSH thesaurus. In this task, the training data consist of a vast collection of MEDLINE abstracts. Each article includes the MeSH terms that the curators used to classify it. It also contains additional metadata such as its unique identifier number (PubMed unique identifier, PMID) used in PubMed (a free search engine for the MEDLINE database), title, journal name, and publication year (see Figure 1). The test data consist of recently published articles that have not been labeled by the curators yet. The participating systems have to find the best MeSH terms and report their answers for the test data.

Biomedical semantic indexing can be defined as a multilabel hierarchical classification problem because each document has to be classified with one or more concepts from a taxonomy. If the taxonomy has a significant number of concepts (more than hundreds), the main challenge is to work with this large number of classes in the classification problem. In the case of the BioASQ challenge, MeSH has a hierarchy with 16 main branches and contains more than 27,000 terms. Some works restrict the scope of MeSH hierarchy using only a particular branch in the MeSH tree (eg, heart diseases) [9] or a subset of terms (generally those appearing in the training collection) [10] to reduce the difficulty of the multilabel classification problem.

### General Architecture

The general architecture of the most state-of-the-art systems comprises 2 differentiated phases: a first phase in which an initial set of MeSH terms is obtained and a second phase that ranks these terms to select the top K that better fit the input document. Several machine-learning techniques have been used such as Support Vector Machines (SVM) [11,12], logistic regression [13], k-nearest neighbors (k-NN) [11,13,14], or a combination of them.

Most previous systems employ either flat classifiers or cascades of classifiers [15]. Flat classifiers [11,16-18] do not take into account the hierarchical relations between the MeSH terms, whereas cascades approaches [19,20] apply a separate classifier top-down for each term. In each term, the method must decide whether to assign the current term to the article being classified or continue descending by the taxonomy and selecting which branches (children) to continue exploring. However, both approaches, flat and cascades, use the BoW (bag-of-words) model to represent the documents. One of the notorious disadvantages of BoW models is that they generate a large number of features (as many as the vocabulary size of the training set), which usually requires prohibitive computation time for practical applications. A possible solution could be the use of feature selection techniques to reduce the number of BoW features. However, these techniques have proved to be inefficient because of the large number of classes (as many as existing terms in MeSH) that must be represented. In other words, as mentioned above, this multilabel classification problem implies more than 20,000 classes (which are the terms stored in MeSH), and it would need to keep at least a few features to represent each class for the classification. Indeed, classifiers used in this problem usually obtain better performance without feature selection [15]. More recently, some works [21,22] use word embedding techniques as an attractive alternative of BoW-based approaches, leading to very large dimensionality reduction and promising results.

Some previous works have implemented different strategies based on the guidelines proposed by human curators to select the most appropriate set of MeSH terms for a given document. However, it is difficult to assess their real utility because human curators, paradoxically, do not always follow their own rules [23].

Table 1 summarizes some of the main systems for the task of biomedical semantic indexing. The underlying characteristics (such as the type of approach: flat vs hierarchical, if the system is based on a search engine, and a brief description of the main techniques used) of these works are presented.

**Table 1:** Main works for biomedical semantic indexing.

System	Type	Guidelines	Search engine	Approach	F1
MTI^a^, Mork et al [14]	Hierarchical	Yes	PubMed	MetaMap, k-NN^b^	0.548
AUTH-Atypon, Papanikolaou et al [12]	Flat	No	No	SVM^c^ with NLP^d^ features	0.578
NCBI^e^, Mao et al [11]	Flat	No	No	SVM + k-NN	0.605
Antinomyra, Liu et al [13]	Flat	No	No	k-NN + logistic regression	0.619
Ribadas et al [18]	Hierarchical	No	No	Bayesian network	0.615
Kosmopoulos et al [21]	Flat	No	No	k-NN + word embeddings	0.57
Peng et al [22]	Flat	No	No	k-NN + word embeddings	0.632

^a^MTI: Medical Text Indexer.

^b^k-NN: k-nearest neighbors.

^c^SVM: Support Vector Machine.

^d^NLP: Natural Language Processing.

^e^NCBI: National Center for Biotechnology Information.

This study is an extension of our earlier work [24] that described our participation on the BioASQ 2016 biomedical semantic indexing (Task 4a). Our main hypothesis is that similar documents should be classified by similar MeSH terms. Although this hypothesis is not new, and whereas most previous works [11,21,22,25] use document similarity by clustering methods such as k-NN algorithm, our approach exploits document similarity computed by an open source search engine, the ElasticSearch tool [26], one of the most efficient document store databases [27]. To the best of our knowledge, very few works have exploited search engines [14,18]. In particular, the work by Ribadas et al [18] used the search engine tool Indri [28], with the drawback of the high computational time needed for its searches.

Although some works [29,30] have applied the semantic similarity between concepts to the biomedical semantic indexing task, very few works have exploited the curators’ guidelines defined by NLM to assign MeSH terms. Our work proposes the implementation of one of the most important annotation rules [1], named “Specific Headings vs Broader Headings,” which had not been considered by any of the previous automatic systems. This rule claims that if 3 MeSH terms are proposed to classify an article and share the same ancestor, then the curator should replace these terms by their lowest common ancestor. To do this, the MeSH thesaurus is represented as a graph database. This model based on graph theory leads to query the thesaurus much faster than using a relation database. It enables to swiftly and effortlessly capture hierarchical relationships such as the shortest path between 2 terms or their lowest common ancestor, which are features very useful to decrease the unnecessary overlapping of MeSH terms when an abstract is classified.

The rest of the paper is organized as follows: first, in the Methods section, we give a description of the datasets used in this study and explain our approach. Then, we report and discuss the results of our method in the Results section. Finally, conclusions and future work are presented.

## Methods

### Objective

The goal of the task was to automatically predict the most descriptive MeSH terms for a given article. The predictions should be compared with MeSH terms that were assigned by human curators. This section describes the MeSH resource, the data, and approach used in this study.

### MeSH

MeSH is a thesaurus of medical concepts, which was created to assist human curators in the task of cataloging the articles in the MEDLINE database. Thus, each MEDLINE document should be represented with a set of MeSH terms that describe its subject topic. MeSH is an annually updated document (eg, 310 new headings were added to MeSH in 2015). The MeSH 2016 version contains a total of 27,883 main terms (also known as headings or descriptors), 82 qualifiers (subheadings), and more than 232,000 supplementary concept records, which represent specific examples of chemicals, diseases, and drug protocols.

**Figure 2:**
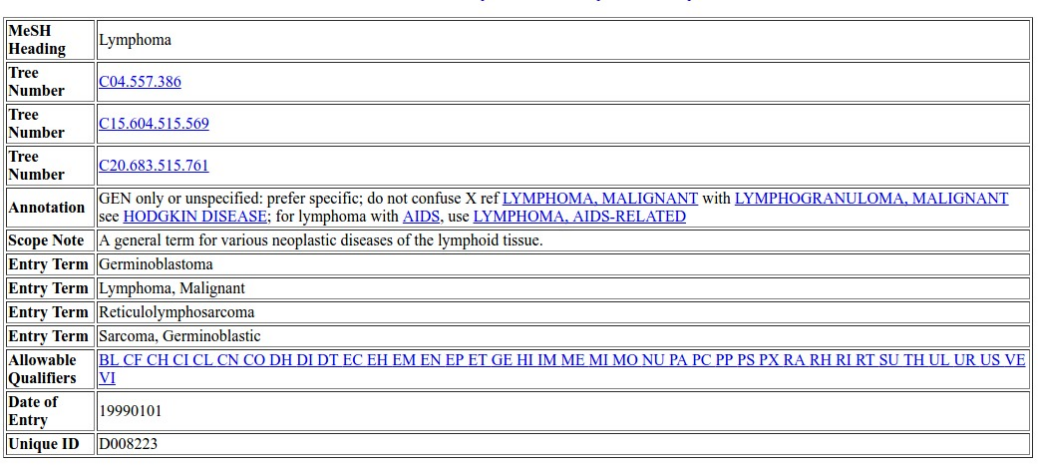
Medical Subject Headings (MeSH) descriptor data for the term "Lymphoma".

In MeSH, most terms contain a short definition, links to related descriptors, a list of synonyms or very similar terms, and a unique alphanumerical ID. Figure 2 shows the content for the term “Lymphoma.” The terms are organized in a hierarchy in which each child can have more than one parent. Therefore, any MeSH term can appear at different branches of the hierarchical structure of MeSH. For example, the term “Lymphoma” belongs to 3 different branches: “Neoplasms [C04],” “Hemic and Lymphatic Diseases [C15],” and “Immunologic Diseases [C20].” The field “Tree Number” represents each possible location of a term in MeSH. Thus, the term “Lymphoma” has 3 tree numbers: C04.557.386, C15.604.515.569, and C20.683.515.761; C stands for Diseases, C04 for Neoplasms, and C04.557 for Neoplasms by Histologic Type; C15 for Hemic and Lymphatic Diseases, C15.604 for Lymphatic Diseases, and C15.604.515 for Lymphoproliferative Disorders; C20 for Immune System Diseases, C20.683 for Immunoproliferative Disorders, and C20.683.515 for Lymphoproliferative Disorders.

### Data

The training data for the BioASQ Task 4a consisted of MEDLINE articles that were manually annotated with MeSH terms by human curators. During the BioASQ 2016 challenge, a test dataset was published each week for the assessment of the participating systems. A total of 15 test datasets were published, which were grouped into 3 different periods (batches). Although the BioASQ challenge ended last May 15, 2016, the test datasets with gold annotations were not released because many articles have not been manually annotated yet.

Two different versions of the training data were provided: (1) Training v.2016a with more than 12 million documents and (2) Training v.2016b with almost 5 million documents from the pool of journals that the BioASQ organizers used to select the articles for the test data. In both datasets, the average number of MeSH terms assigned to an article was 12 to 13.

In our previous work [24], we performed several experiments using each of the 2 training datasets, which led to the conclusion that they did not make a significant difference on the performance of our system. For this reason, we decided to only use the largest dataset (Training v.2016a ) to perform all of the experiments described in this new work (see the Results section). Moreover, to optimize the best setting of our approach, we randomly chose 1099 documents from the training dataset and separated them for development set.

**Figure 3:**
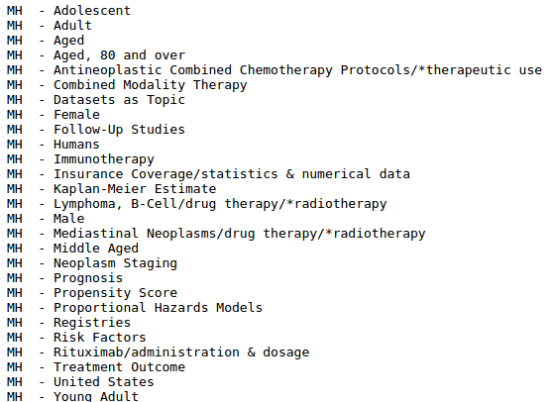
MeSH terms for the abstract with Pubmed unique identifier (PMID)=26852276.

As mentioned before, no test datasets with gold standard annotations were released. However, to perform a transparent and consistent evaluation of our work, we developed a script that obtains the MeSH terms for all abstracts in the test batches of the 2016 BioASQ. For each test document, the script obtains its PMID and then generates a query for searching it in PubMed. If the PMID exists in MEDLINE, PubMed returns a structured document containing the metadata for this abstract, among them its MeSH labels (see Figure 3), collected by the script using a regular expression. Finally, the labels are also searched in the MeSH resource to obtain their corresponding MeSH identifiers. In this way, we obtained the same 15 test datasets used in the 2016 BioASQ edition. Table 2 shows the size of the different datasets used in this study.

**Table 2:** Size of datasets (number of documents).

Dataset	Documents, n
Training	10,099,281
Development	1099
Test	13,936

### Indexing Documents and Query (Test Document) Using ElasticSearch

Our approach relies on the assumption that similar documents should be classified by similar MeSH terms. Previous research has generally used document clustering techniques, such as the k-NN algorithm, to obtain the similar documents for a given test document. Instead of using k-NN, we proposed the use of an open source search engine, ElasticSearch, to retrieve a set of similar documents for each test document.

**Figure 4:**
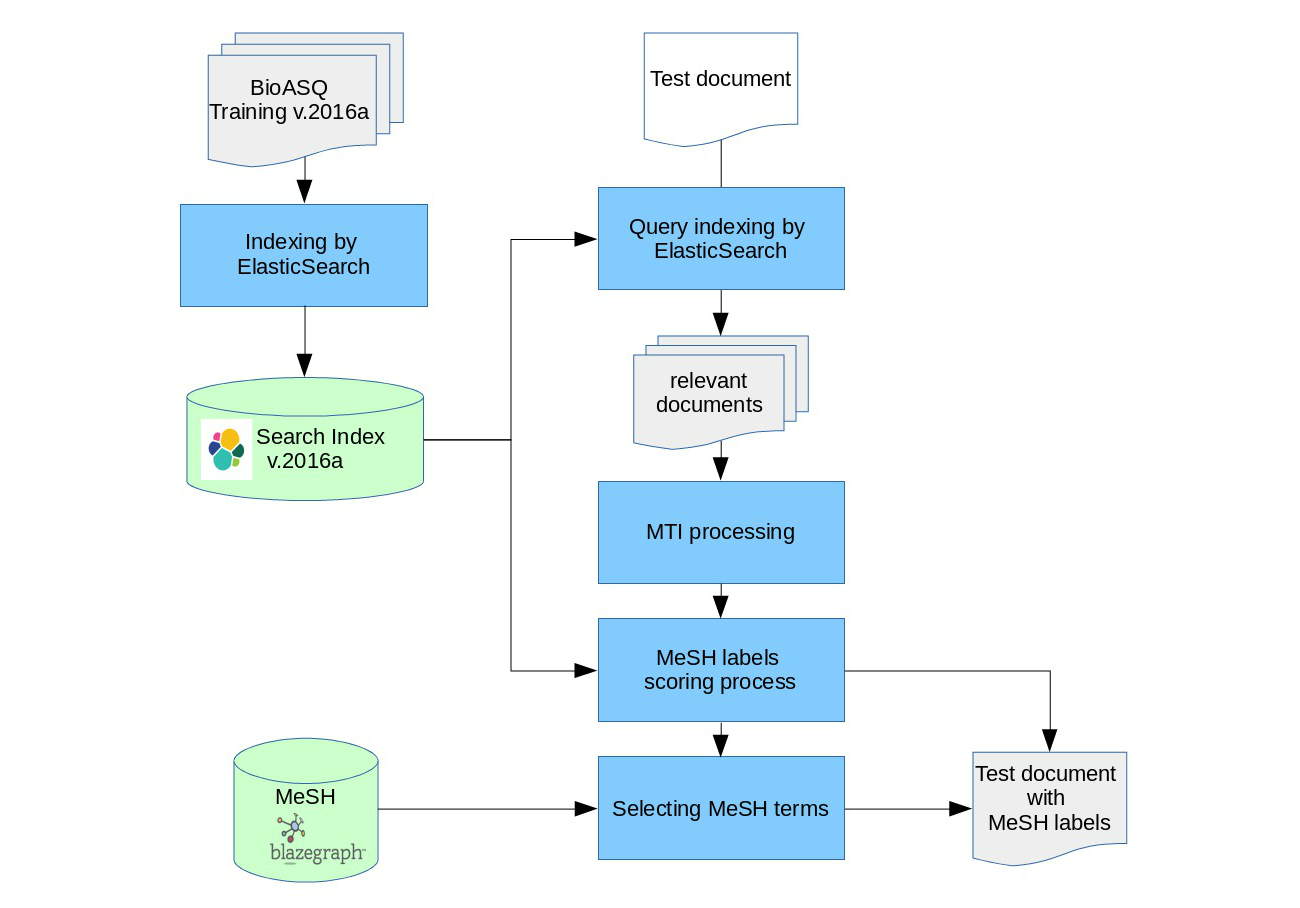
Architecture of our system.

Figure 4 shows the main steps of our approach. ElasticSearch was used to index all the documents of the training dataset (Training v.2016a). Each training document was stored along with its corresponding MeSH terms. Each test document was also represented as a query, which was fired against the index built from the training dataset. Then, ElasticSearch should return the most relevant (similar) documents to the query (the test document). Finally, our system initially assigns it all the MeSH terms of the similar documents retrieved by ElasticSearch for this document.

Below we explain in detail how the index was constructed and how a query (a test document) could be compared against this index to recover the most relevant (similar) documents.

The core of ElasticSearch is Apache Lucene, a free, open-source, and *de facto* standard retrieval software library (by The Apache Software Foundation). The efficiency of Lucene is because it searches on index instead of searching the text directly. Moreover, the index is stored in the main memory.

**Figure 5:**
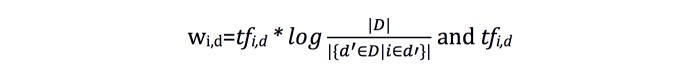
The element wi,d is the frequency of the term i in the document d.

Lucene is based on the well-known and commonly used vector space model (VSM) for information retrieval. This model allows us to represent documents as vectors, where each position in the vector represents a specific term (typically terms are single words), and the value at that position denotes the weight of that term. There are several different ways of computing these values, being the most known term frequency-inverse document frequency (tf-idf) weighting. In this model, a given document *d* is represented as a vector *v*_d_*=[w*_1,d_*, w*_2,d_*,…, w*_N,d_*]*, where w_i,d_ represents the frequency of the term *i* in the document *d*, *D* is the set of all documents, and *|{d'Є D|I Є d'}|* is the number of documents containing the term *I* (see Figure 5).

**Figure 6:**
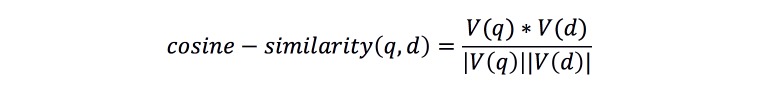
Cosine similarity between a document d and a query w, where V(q).V(d) is the dot product of their vectors, and |V(q)| and |V(d)| are their Euclidean norms.

In short, VSM represents documents and queries as weighted vectors, where each dimension refers to an index term and its value is its tf-idf value. To assess the relevance of a document *d* for a given query *q*, VSM calculates the cosine similarity of their vectors (see Figure 6). Therefore, the basic idea behind VMS is that the more frequent a term is in a document relative to its frequency in the whole collection of documents, the more relevant that document is to the query.

Another important advantage of ElasticSearch is its capacity to create distributed and scalable systems by specifying only the configuration of the hierarchy of nodes. Thus, ElasticSearch is self-managed to maintain better fault tolerance and load distribution. In 2014, an empirical evaluation study about the effectiveness of the current databases demonstrated that ElasticSearch achieved the best performance compared with other document store databases [27]. This is because ElasticSearch uses the main memory and compresses documents, thereby improving retrieval time. Moreover, another main challenge of the task is to manage the great amount of documents that have to be indexed. Thanks to its horizontal scalability (ie, the possibility of adding more storage and processing power), ElasticSearch is able to index large collections of documents such as the MEDLINE database.

In this study, ElasticSearch (version 5.0) was installed on an Ubuntu 16.04 server with 24 GB of RAM and 500 GB of disk space. It took 10,264.07 seconds to index all the training dataset (ie, an average of 1.02 milliseconds per document). The training dataset (Training v.2016a) consists of a total of 10,100,380 documents, with an average size of 2.1 KB per document.

#### MTI Processing

The Medical Text Indexer (MTI) [14] is a tool developed by NLM and is considered as a baseline system for the task, which provides a preliminary annotation of the articles. MTI is based on a combination of MetaMap- [31] and PubMed-related citations to recognize MeSH terms that are then clustered and ranked by a k-NN algorithm. Given a document, MTI uses MetaMap to find its concepts. The UMLS (Unified Medical Language System) concepts found by MetaMap are restricted to MeSH by a combination of synonym and interconcept relations, and mappings. MTI also obtains a second list of MeSH terms by obtaining similar documents for the input document. To do this, MTI uses the list of PubMed-related citations provided by the PubMed system. Then, the MeSH terms of these similar documents are also extracted. Finally, MTI clusters both lists of MeSH terms into a single list. Terms are clustered by a k-NN algorithm and ranked according to the product of the frequency and the MeSH tree depth of each term. MTI also includes a postprocessing phase that implements a set of filtering rules from the NLM guidelines. For instance, it contains a list of triggers that activate one or more MeSH tags and that comes mainly from the NLM guidelines, in the way of rules such as “if XXXX appears in the text then you should tag as AAAA.”

As it was mentioned before, our system initially considered the set of MeSH terms from the relevant documents retrieved by ElasticSearch for a given test document. Then, that set was further extended with those terms provided by the MTI tool.

#### MeSH Labels Scoring Process

**Figure 7:**
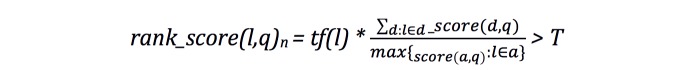
Scoring function to rank Medical Subject Headings (MeSH) term.

In the previous two sections, we described how an initial set of MeSH terms is proposed by ElasticSearch and later extended by the MTI tool, for a given test document. In this section, we introduce a new scoring function to rank the MeSH terms for a given test document (represented as a query *q*). The basic idea behind this scoring function is the more number of times a MeSH term appears in the set of more relevant documents for a given test document (query), the more significant that term is to this test document. The scoring function (see Figure 7) for a MeSH term *l* and a test document *q* considers the following parameters:

*tf(l)*: the frequency of the MeSH term *l* in the set of retrieved documents by ElasticSearch for the document *q (query)*.

*Σ*_d:lЄd_*_score (d, q)* is the sum of all scores of the relevant documents to the query *q*, which also contain the MeSH term *l*. As mentioned before, ElasticSearch uses the cosine similarity function to obtain the score between a document and a query. We normalized the sum of all scores because some documents may present a large number of MeSH terms, whereas others very few. To do that, we divided it by the maximum score of the relevant documents containing the term *l*.

*T* is a real positive value that represents the minimum threshold for the scores of the MeSH terms. That is, only the MeSH terms whose scores are greater than *T* finally will be selected for cataloging the test document *q*.

#### Selecting MeSH Terms by Exploiting a Graph Database

In this point, we already have a set of ranked MeSH terms for a given test document.

In the last phase, we implemented a heuristic based on the guidelines of human annotators [1] to classify MEDLINE articles. In particular, the implemented rule claimed that if an abstract had 3 or more MeSH terms sharing some ancestor, then the curators should replace these 3 terms by their lowest common ancestor.

Our hypothesis here was that representing the MeSH thesaurus as a graph would let to query the MeSH thesaurus much faster than when using its original format. By using well-known graph search algorithms such as depth-first search, the model graph enabled to rapidly and easily capture hierarchical relationships such as the shortest path between 2 terms or their lowest common ancestor. Knowing these hierarchical relationships allowed us to find the most appropriate MeSH terms for a given abstract, decreasing the possible overlapping among them, as the NLM recommends.

BlazeGraph [32] is a graph database with support for Java APIs (Application Program Interface) and standardized query languages for graphs, such as SPARQL (Protocol and RDF Query Language). An important advantage of BlazeGraph is that it processes large graphs in near-real time by its GPU (Graphical Processor Unit) acceleration achieving better processing time than CPU (Central Processing Unit) technologies or other graph databases based on key values.

NLM provides a beta version of the MeSH thesaurus in RDF (Resource Description Framework), a standard format for linked open data. This RDF version of MeSH can be loaded into BlazeGraph using the dotNetRDF API, a free and open-source project for working with RDF, SPARQL, and the Semantic Web.

We also developed an algorithm that, given an input document, traverses each of the MeSH terms proposed in the previous step and searches its ancestors by querying the graph database of MeSH with the depth-first search algorithm. Finally, when our algorithm finds out that 3 or more of its MeSH terms share the same ancestor, it replaces them by their lowest common ancestor.

**Figure 8:**
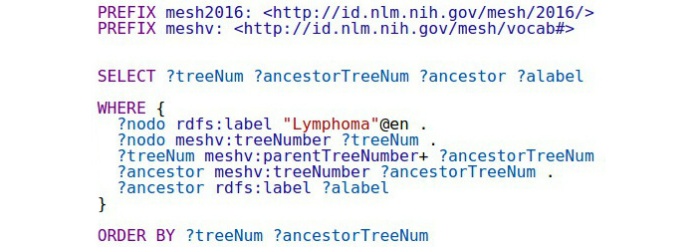
BlazeGraph query to obtain the ancestors of the term "Lymphoma".

**Figure 9:**
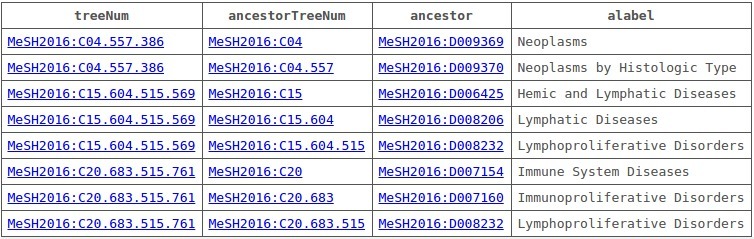
List of ancestors for the term "Lymphoma" provided by BlazeGraph.

Initially, we restricted the search to a given depth of ancestors, that is, pruning the search subtree below to a given height. However, because the maximum depth is relatively small (consisting only of 9 levels, with an average depth of approximately 4.5 levels), we decided to explore the complete tree of ancestors for each term. Figure 8 shows the query used by ElasticSearch to retrieve all ancestors of the term “Lymphoma.” The output of this query is shown in Figure 9 where the term “Lymphoma” is in 3 different branches of the MeSH thesaurus: C04-Neoplasms, C15-Hemic and Lymphatic Diseases, and C20-Immune System Diseases. M

Table 3 shows the list of MeSH terms proposed by our system for the article with PMID=25676421. The first column contains the MeSH terms after applying our script to replace the terms (3 or more) sharing the same ancestor, whereas the second one contains the MeSH terms proposed by using only ElasticSearch and the score function. For example, the terms “Lymphoma, B-Cell,” “Ataxia Telangiectasia,” and “Lymphoma” were substituted by their lowest common ancestor “Immune System Diseases.”

Table 4 shows the comparison of search times for 3 different MeSH terms. The reader can see that the 3 searches on the MeSH thesaurus stored into a graph database are significantly faster than the same searches on the RDF format.

**Table 3:** MeSH (Medical Subject Headings) terms proposed by our system for the article with PMID (PubMed unique identifier)=25676421.

MeSH^a^ exploiting the hierarchy of MeSH	MeSH terms
Ataxia Telangiectasia Mutated Proteins	Ataxia
	Telangiectasia Mutated
	Proteins
B-Lymphocytes	B-Lymphocytes
Cell Cycle Proteins	Cell Cycle Proteins
DNA-Binding Proteins	DNA-Binding Proteins
Humans	Humans
Protein-Serine-Threonine Kinases	Protein-Serine-Threonine
	Kinases
Animals	Animals
Genomic Instability	Genomic Instability
Mice, Knockout	Mice, Knockout
Cyclin D1 Mice In Situ Hybridization, Fluorescence	Cyclin D1 In Situ
	Hybridization, Fluorescence
Immune System Diseases	Lymphoma, B-Cell
	Ataxia Telangiectasia
	Lymphoma

^a^MeSH: Medical Subject Headings.

**Table 4:** Comparison of search times on the Resource Description Framework (RDF) format and the graph database of the MeSH (Medical Subject Headings) thesaurus.

MeSH^a^ terms	RDF^b^ in ms^c^	Graph database in ms
Lymphoma, B-Cell	193.39	112
Cyclin D1	210.44	100
Mice, Knockout	239.86	130

^a^MeSH: Medical Subject Headings.

^b^RDF: Resource Description Framework.

^c^ms: milliseconds.

## Results

### Design of the Experiments

This section conducts an exhaustive set of experiments, where different parameters and options are evaluated on the development dataset to determine the best setting for our system, which will finally be evaluated on the test datasets.

In BioASQ, the performance of the participating systems is evaluated using standard IR measures (eg, precision, recall, and F1), as well as hierarchical variants of them, such as the lowest common ancestor Precision (LCA_P), Recall (LCA_R) and F-measure (LCA-F). The reader can find a detailed explication of these measures in the article [33]. The HEMKit software [34], a tool that implements these measures and lets to easily evaluate the results of different experiments, was used to provide the scores.

Our experiments aimed to answer the following questions:

What is the effect of the number of relevant documents retrieved by ElasticSearch? It is expected that the more documents the search engine obtains, the higher the recall and the lower the precision of our system. We experimented with different number of relevant documents to obtain the best balance between precision and recall, that is, the best F1. In particular, we tried with 10, 20, 30, 40, and 50 documents.

What is the best threshold T that we should consider in our scoring function? Higher values of this threshold should provide a high precision but with a significant decrease of recall. Our objective was to determine the optimum value of this parameter T, that is, that value that obtains the highest F1.

Does the use of the hierarchical structure of MeSH improve the performance of our system? In particular, we assess whether the strategy of replacing terms sharing the same ancestor by their lowest common ancestor helped to improve the performance.

### Experiment With/Without exploiting MeSH Hierarchical Structure

Tables 5 and 6 show the results exploiting the hierarchical structure of MeSH and without it, respectively. Each experiment is represented with the label *Elastic-X-T*, where X refers to the number of relevant documents retrieved by ElasticSearch and T to the threshold for our scoring function.

We tried with different number of retrieved relevant documents; in particular, the parameter X could take the following values: 10, 20, 30, 40, and 50. Although increasing the number of retrieved relevant documents achieves to improve the recall, it has a very negative effect on the precision of our system. Indeed, the best F1 (if we do not use the structure of MeSH, we obtain F1=0.70) is obtained with the lowest number of retrieved relevant documents regardless the value of the threshold T (see Tables 5 and 6). Therefore, we can conclude that the best value of X is 10. For values less than 10, the recall decreases significantly. In other words, the system achieves better performance if the search engine is set up to return at least 10 documents.

To assess the effect of the threshold T on the performance of our system, we tried with different values. Tables 5 and 6 show the results for values of T in range (0,9). The reader can see that, in general, the greater the value of the parameter T, the higher the precision, and also the maximum F1. However, the recall decreases when increasing the value of T. Any value lower than 1 achieves a very high recall but very low precision because the system would return all MeSH terms obtained by ElasticSearch along with those provided by the MTI tool, without applying any filter. That is, if the value of T is lower than 1, the scoring function does not rule out any term from the initial set of MeSH terms proposed by ElasticSearch and MTI. On the other hand, for values of T up to 5, the performance begins to drop. In general, best results are obtained for T equal to 5.

**Table 5:** Experimental results on our development dataset exploiting the hierarchical structure of Medical Subject Headings (MeSH).

Elastic-X-T	Precision	Recall	F1	LCA-P^a^	LCA-R^b^	LCA-F^c^
Elastic-10-0	0.3021	0.8784	0.4386	0.2061	0.6046	0.3006
Elastic-10-1.5	0.6290	0.6213	0.6039	0.4146	0.3979	0.3880
Elastic-10-2.5	0.6599	0.6214	0.6179	0.4376	0.3981	0.3982
Elastic-10-4	0.7371	0.6130	0.6466	0.4936	0.3927	0.4179
Elastic-10-5	0.7898	0.5987	0.6576	0.5316	0.3843	0.4256
Elastic-10-6	0.7434	0.6107	0.6475	0.4986	0.3914	0.4185
Elastic-10-7	0.7904	0.5980	0.6573	0.5321	0.3840	0.4255
Elastic-10-8	0.7968	0.5937	0.6566	0.5372	0.3415	0.4254
Elastic-10-9	0.7910	0.5976	0.6571	0.5325	0.3838	0.4255
Elastic-20-0	0.2174	0.9248	0.3441	0.1564	0.6530	0.2475
Elastic-20-1.5	0.5268	0.6303	0.5546	0.3396	0.4045	0.3542
Elastic-20-2.5	0.5723	0.6331	0.5803	0.3709	0.4044	0.3703
Elastic-20-4	0.6266	0.6332	0.6080	0.4108	0.4047	0.3901
Elastic-20-5	0.6879	0.6294	0.6350	0.4580	0.4037	0.4100
Elastic-20-6	0.6413	0.6333	0.6150	0.4221	0.4054	0.3953
Elastic-20-7	0.6914	0.6296	0.6367	0.4605	0.4039	0.4112
Elastic-20-8	0.7104	0.6279	0.6441	0.4755	0.4041	0.4173
Elastic-20-9	0.6945	0.6299	0.6383	0.4630	0.4043	0.4124
Elastic-30-0	0.1790	0.9434	0.2945	0.1331	0.6770	0.2185
Elastic-30-1.5	0.4776	0.6388	0.5290	0.3040	0.4108	0.3359
Elastic-30-2.5	0.5231	0.6341	0.5537	0.3374	0.4076	0.3537
Elastic-30-4	0.5652	0.6323	0.5766	0.3668	0.4046	0.3685
Elastic-30-5	0.6200	0.6354	0.6067	0.4063	0.4060	0.3888
Elastic-30-6	0.5831	0.6332	0.5864	0.3786	0.4050	0.3748
Elastic-30-7	0.6256	0.6359	0.6096	0.4104	0.4069	0.3911
Elastic-30-8	0.6506	0.6369	0.6217	0.4293	0.4070	0.3993
Elastic-30-9	0.6302	0.6364	0.6120	0.4139	0.4069	0.3928
Elastic-40-0	0.1555	0.9532	0.2621	0.1184	0.6924	0.1988
Elastic-40-1.5	0.4412	0.6473	0.5081	0.2801	0.4166	0.3227
Elastic-40-2.5	0.4915	0.6383	0.5366	0.3145	0.4106	0.3417
Elastic-40-4	0.5302	0.6343	0.5582	0.3404	0.4077	0.3556
Elastic-40-5	0.5755	0.6359	0.5840	0.3726	0.4069	0.3726
Elastic-40-6	0.5472	0.6356	0.5682	0.3521	0.4073	0.3618
Elastic-40-7	0.5819	0.6370	0.5878	0.3777	0.4083	0.3758
Elastic-40-8	0.6073	0.6374	0.6011	0.3959	0.4077	0.3847
Elastic-40-9	0.5870	0.6374	0.5908	0.3813	0.4082	0.3777
Elastic-50-0	0.1395	0.9603	0,239	0.1082	0.7045	0.1846
Elastic-50-1.5	0.4161	0.6542	0.4930	0.2628	0.4226	0.3127
Elastic-50-2.5	0.4669	0.6445	0.5239	0.2965	0.4151	0.3328
Elastic-50-4	0.5008	0.6392	0.5431	0.3192	0.4112	0.3452
Elastic-50-5	0.5447	0.6357	0.5670	0.3500	0.4081	0.3610
Elastic-50-6	0.5192	0.6390	0.5538	0.3324	0.4096	0.3518
Elastic-50-7	0.5507	0.6361	0.5702	0.3548	0.4086	0.3637
Elastic-50-8	0.5767	0.6366	0.5849	0.3734	0.4074	0.3734
Elastic-50-9	0.5560	0.6360	0.5733	0.3585	0.4081	0.3656

^a^LCA-P: lowest common ancestor Precision.

^b^LCA-R: lowest common ancestor Recall.

^c^LCA-F: lowest common ancestor F-measure.

The exploitation of the hierarchical structure of MeSH does not improve the results; on the contrary, the recall is dropped almost by 5% (see Tables 5 and 6). Therefore, we can conclude that the strategy of replacing terms sharing the same ancestor by their lowest common ancestor does not increase the results. A possible explication for this fact could be that human curators do not to follow the annotation guidelines.

The pattern of the hierarchical scores (LCA-P, LCA-R, and LCA-F1) according to the different parameters is very similar to the behavior of the flat scores. That is, the best hierarchical scores are usually obtained using the lowest number of retrieved relevant documents and the threshold of the score function equal to 8. Likewise in the flat setting, the rule of replacing 3 or more MeSH terms by their lowest common ancestor does not seem to improve the results.

### Experiments on BioASQ 2016 Test Dataset

Finally, we ran the best setting (X=10, T=5) on the test datasets. Tables 7 and 8 show the results of this setting exploiting the structure of MeSH and those without it, respectively. As in the development dataset, the performance is better if we do not use the structure of MeSH.

As mentioned above, the MTI system is considered the baseline for the task. Table 9 shows the results achieved by MTI on each test set published in the 2016 BioASQ. The top F1 is 0.5196 and top LCA-F is 0.4807.

Table 10 shows the temporary scores of the best systems in BioASQ Task 4a. The reader can see that the best F1 rates are between 58% and 65%, the best recall between 54% and 60%, and the best precision between 60% and 72%, depending on the batch. Our approach that does not exploit the hierarchical structure of MeSH seems to obtain better performance than the top systems (see Table 8). Our best F1 is 0.70 (batch 1, week 1). On the other hand, if our system uses the hierarchical relations of MeSH to select the best set of terms to label a given article, this obtains an F1 of 0.67, also better than the top F1 (0.61) of the best systems. Therefore, we can conclude that our approach achieves to overcome the top participating systems at the BioASQ 2016.

**Table 6:** Experimental results on our development dataset without using the hierarchical structure of Medical Subject Headings.

Systems	Precision	Recall	F1	LCA-P^a^	LCA-R^b^	LCA-F^c^
Elastic-10-0	0.4201	0.6273	0.4858	0.2678	0.4074	0.3104
Elastic-10-1.5	0.5737	0.7755	0.6439	0.3749	0.5260	0.4258
Elastic-10-2.5	0.6128	0.7598	0.6602	0.4017	0.5151	0.4374
Elastic-10-4	0.7102	0.7125	0.6927	0.4701	0.4812	0.4599
Elastic-10-5	0.7724	0.6755	0.7010	0.5141	0.4515	0.4636
Elastic-10-6	0.7178	0.7074	0.6935	0.4761	0.4773	0.4605
Elastic-10-7	0.7731	0.6746	0.7007	0.5149	0.4508	0.4634
Elastic-10-8	0.7803	0.6684	0.6997	0.5204	0.4456	0.4624
Elastic-10-9	0.7738	0.6740	0.7005	0.5154	0.4505	0.4634
Elastic-20-0	0.3498	0.6548	0.4413	0.2229	0.4274	0.2829
Elastic-20-1.5	0.4263	0.8527	0.5559	0.2821	0.5856	0.3723
Elastic-20-2.5	0.4859	0.8324	0.5982	0.3191	0.5702	0.3982
Elastic-20-4	0.5631	0.8008	0.6458	0.3678	0.5459	0.4280
Elastic-20-5	0.6433	0.7643	0.6820	0.4230	0.5213	0.4534
Elastic-20-6	0.5822	0.7926	0.6547	0.3811	0.5406	0.4345
Elastic-20-7	0.6479	0.7627	0.6838	0.4265	0.5203	0.4549
Elastic-20-8	0.6713	0.7515	0.6917	0.4434	0.5131	0.4609
Elastic-20-9	0.6518	0.7608	0.6852	0.4296	0.5195	0.4563
Elastic-30-0	0.3141	0.6747	0.4152	0.2023	0.4444	0.2690
Elastic-30-1.5	0.3538	0.8876	0.4950	0.2380	0.6146	0.3362
Elastic-30-2.5	0.4165	0.8668	0.5492	0.2760	0.5972	0.3686
Elastic-30-4	0.4769	0.8429	0.5956	0.3124	0.5773	0.3959
Elastic-30-5	0.5528	0.8115	0.6428	0.3602	0.5541	0.4254
Elastic-30-6	0.5016	0.8336	0.6113	0.3281	0.5705	0.4059
Elastic-30-7	0.5602	0.8087	0.6466	0.3652	0.5524	0.4281
Elastic-30-8	0.5913	0.7952	0.6623	0.3860	0.5430	0.4388
Elastic-30-9	0.5657	0.8067	0.6494	0.3690	0.5508	0.4300
Elastic-40-0	0.2905	0.6895	0.3962	0.1881	0.4562	0.2581
Elastic-40-1.5	0.3086	0.9071	0.4508	0.2112	0.6319	0.3106
Elastic-40-2.5	0.3710	0.8862	0.5110	0.2484	0.6135	0.3460
Elastic-40-4	0.4200	0.8675	0.5534	0.2777	0.5979	0.3710
Elastic-40-5	0.4895	0.8416	0.6054	0.3200	0.5770	0.4020
Elastic-40-6	0.4469	0.8591	0.5740	0.2942	0.5909	0.3834
Elastic-40-7	0.4980	0.8383	0.6106	0.3254	0.5752	0.4054
Elastic-40-8	0.5327	0.8242	0.6321	0.3471	0.5639	0.4187
Elastic-40-9	0.5052	0.8359	0.6152	0.3300	0.5733	0.4083
Elastic-50-0	0.2719	0.7006	0.3803	0.1776	0.4651	0.2496
Elastic-50-1.5	0.2769	0.9204	0.4168	0.1925	0.6458	0.2911
Elastic-50-2.5	0.3379	0.9003	0.4805	0.2287	0.6261	0.3282
Elastic-50-4	0.3791	0.8845	0.5192	0.2527	0.6118	0.3504
Elastic-50-5	0.4420	0.8615	0.5718	0.2904	0.5926	0.3813
Elastic-50-6	0.4065	0.8757	0.5425	0.2694	0.6042	0.3643
Elastic-50-7	0.4513	0.8578	0.5783	0.2961	0.5901	0.3854
Elastic-50-8	0.4876	0.8445	0.6043	0.3184	0.5793	0.4010
Elastic-50-9	0.4594	0.8555	0.5841	0.3012	0.5883	0.3890

^a^LCA-P: lowest common ancestor Precision.

^b^LCA-R: lowest common ancestor Recall.

^c^LCA-F: lowest common ancestor F-measure.

**Table 7:** Results on the biomedical semantic indexing and question answering 2016 test datasets (exploiting the Medical Subject Headings hierarchy.

Test		Precision	Recall	F1	LCA-P^a^	LCA-R^b^	LCA-F1^c^
**Batch1**							
	Week1	0.705	0.619	0.635	0.470	0.389	0.406
	Week2	0.717	0.627	0.646	0.476	0.397	0.413
	Week3	0.701	0.625	0.635	0.467	0.395	0.407
	Week4	0.725	0.613	0.643	0.486	0.385	0.410
	Week5	0.707	0.624	0.638	0.474	0.398	0.410
**Batch2**							
	Week1	0.695	0.633	0.637	0.457	0.398	0.405
	Week2	0.713	0.637	0.649	0.467	0.410	0.412
	Week3	0.691	0.637	0.673	0.464	0.402	0.410
	Week4	0.676	0.659	0.641	0.446	0.420	0.4120
	Week5	0.686	0.660	0.648	0.448	0.414	0.409
**Batch3**							
	Week1	0.701	0.625	0.639	0.461	0.403	0.410
	Week2	0.698	0.652	0.648	0.457	0.408	0.407
	Week3	0.694	0.641	0.641	0.447	0.406	0.405
	Week4	0.429	0.513	0.399	0.284	0.264	0.258
	Week5	0.674	0.660	0.640	0.447	0.419	0.409

^a^LCA-P: lowest common ancestor Precision.

^b^LCA-R: lowest common ancestor Recall.

^c^LCA-F: lowest common ancestor F-measure.

**Table 8:** Results on the biomedical semantic indexing and question answering 2016 test datasets (without exploiting the Medical Subject Headings hierarchy).

Test		Precision	Recall	F1	LCA-P^a^	LCA-R^b^	LCA-F1^c^
**Batch1**							
	Week1	0.665	0.753	0.687	0.438	0.503	0.452
	Week2	0.674	0.767	0.700	0.441	0.513	0.460
	Week3	0.661	0.755	0.684	0.437	0.509	0.453
	Week4	0.683	0.749	0.697	0.451	0.502	0.460
	Week5	0.667	0.757	0.690	0.438	0.509	0.455
**Batch2**							
	Week1	0.655	0.755	0.681	0.427	0.501	0.445
	Week2	0.669	0.758	0.692	0.427	0.508	0.454
	Week3	0.653	0.757	0.681	0.433	0.509	0.452
	Week4	0.639	0.764	0.674	0.420	0.516	0.445
	Week5	0.643	0.797	0.692	0.417	0.531	0.451
**Batch3**							
	Week1	0.666	0.746	0.684	0.437	0.512	0456
	Week2	0.654	0.774	0.690	0.421	0.517	0.448
	Week3	0.655	0.754	0.680	0.426	0.507	0.446
	Week4	0.390	0.475	0.410	0.254	0.311	0.268
	Week5	0.663	0.770	0.672	0.416	0.516	0.442

^a^LCA-P: lowest common ancestor Precision.

^b^LCA-R: lowest common ancestor Recall.

^c^LCA-F: lowest common ancestor F-measure.

**Table 9:** Baseline results provided by the Medical Text Indexer (MTI) tool. These results were taken from the biomedical semantic indexing and question answering website.

Test		Precision	Recall	F1	LCA-P^a^	LCA-R^b^	LCA-F1^c^
**Batch1**							
	Week1	0.558	0.516	0.493	0.498	0.462	0.463
	Week2	0.550	0.514	0.487	0.516	0.478	0.480
	Week3	0.553	0.537	0.507	0.499	0.467	0.465
	Week4	0.568	0.505	0.482	0.507	0.455	0.464
	Week5	0.558	0.508	0.484	0.504	0.474	0.473
**Batch2**							
	Week1	0.546	0.520	0.493	0.495	0.473	0.467
	Week2	0.544	0.520	0.492	0.497	0.471	0.469
	Week3	0.558	0.526	0.500	0.503	0.470	0.470
	Week4	0.549	0.516	0.491	0.487	0.452	0.449
	Week5	0.532	0.551	0.519	0.480	0.487	0.467
**Batch3**							
	Week1	0.515	0.459	0.444	0.492	0.441	0.449
	Week2	0.543	0.484	0.466	0.493	0.455	0.455
	Week3	0.580	0.502	0.486	0.512	0.457	0.466
	Week4	0.545	0.522	0.494	0.496	0.481	0.469
	Week5	0.536	0.517	0.496	0.499	0.473	0.466

^a^LCA-P: lowest common ancestor Precision.

^b^LCA-R: lowest common ancestor Recall.

^c^LCA-F: lowest common ancestor F-measure.

**Table 10:** Results of the top systems in biomedical semantic indexing and question answering (BioASQ) task 4a. These scores were taken on December 5 from the BioASQ website.

Batch	System	Week	Number of annotated articles	Total of articles	Precision	Recall	F1
1	MeSHLabeler	1	1853	3740	0.626	0.521	0.513
	MeSHLabeler	2	1578	2872	0.625	0.515	0.506
	MeSHLabeler	3	1115	2599	0.602	0.519	0.515
	MeSHLabeler-1	4	1436	3294	0.649	0.496	0.495
	MTI	5	1181	3210	0.558	0.508	0.484
2	MTI	1	1080	3212	0.546	0.520	0.493
	MeSHLabeler-2	2	901	3213	0.630	0.505	0.499
	MeSHLabeler-2	3	850	2831	0.642	0.521	0.516
	MTI	4	800	3111	0.549	0.516	0.491
	MeSHLabeler	5	688	2470	0.615	0.538	0.526
3	MeSHLabeler	1	305	2994	0.637	0.462	0.462
	MeSHLabeler	2	507	3044	0.6449	0.4851	0.4825
	MeSHLabeler	3	501	3351	0.6544	0.4991	0.4956
	MeSHLabeler	4	514	2630	0.6312	0.5098	0.5012
	MeSHLabeler	5	627	3130	0.5017	0.5119	0.6135

## Discussion

### Principal Findings

Our approach relies on the assumption that similar documents should be classified by similar MeSH terms. Previous works have already applied a k-NN approach for obtaining the set of similar document for a given test document. Our previous work [24] and this study are the first efforts to explore the document similarity using the search engine ElasticSearch instead of k-NN. ElasticSearch is one of the most efficient document-based database. Given a test document, this is represented as a query, which is executed in the search engine, returning the documents more relevant (similar) to the query. Then, our system proposes the MeSH of all these documents as the initial set of MeSH terms for the test document and extends this set with the MeSH terms proposed by the MTI tool. Finally, the system uses a scoring function to determine the best set of MeSH terms for a given article. Those MeSH terms that achieve a higher score than a given threshold are finally selected. The experiments show that the best results are obtained when the number of retrieved relevant documents by ElasticSearch is small (10) and the threshold for the scoring function is equal to 5.

### Comparison With Prior Work

Our approach seems to provide better results than the top systems in BioASQ 2016. We note that our results are not immediately comparable with those reported by the BioASQ challenge because we have used a different test dataset. However, we think that it is a reasonable evaluation while no official test datasets are available. Moreover, our development test datasets are available at our webpage [35] to facilitate reproducible research, objective assessment, and further analysis.

In addition, we implement one of the guidelines established by human curators to classify MEDLINE abstracts. To do this, we store the MeSH thesaurus into a graph-based database by using the BlazeGraph tool. The main advantage of using a graph structure is the possibility to use algorithms well known in graph theory (such as depth-first search) to extract subgraphs satisfying a given query. In particular, the graph is visited with the objective to determine whether 3 or more MeSH terms assigned to a given article share the same ancestor. In this case, this lowest common ancestor should substitute them. Contrary to expectations, the system produces worse results if this rule is applied. This may be because human curators do not always follow the recommendations to catalog MEDLINE abstracts.

### Limitations

Although the results are better when we do no exploit the hierarchy of MeSH, we think that the graph database version of MeSH is a promising resource that will allow us to implement other guidelines or strategies to select the most appropriate MeSH terms for representing a given article.

### Conclusions

Semantic indexing of MEDLINE articles is a manual, laborious task, which could be helped by information technology.

As future steps, we also plan to determine semantic similarity between documents using word embeddings [36] instead of the well-known and commonly used VSM for information retrieval. This approach has already been exploited by Liu et al [21] and Kosmopoulos et al [22]. Unlike these works, based on the use of k-NN for obtaining the set of similar documents, our approach will continue using ElasticSearch as search engine and our graph database format of MeSH. We also plan to explore deep learning methods (such as Convolutional Neural Networks) for supporting the automatic classification of MEDLINE abstracts.

## Abbreviations

BioASQ: biomedical semantic indexing and question answering challenge
BoW: bag-of-words
F1: F-measure
k-NN: k-nearest neighbors
LCA: lowest common ancestor
LCA-P: lowest common ancestor Precision
LCA-R: lowest common ancestor Recall
LCA-F: lowest common ancestor F-measure
MeSH: Medical Subject Headings
MTI: Medical Text Indexer
NCBI: National Center for Biotechnology Information
NLM: National Library of Medicine
NLP: Natural Language Processing
PMID: PubMed unique identifier
RDF: Resource Description Framework.
SVM: Support Vector Machine
UMLS: Unified Medical Language System
VSM: vector space model
